# Spatio-Temporal Updating in the Left Posterior Parietal Cortex

**DOI:** 10.1371/journal.pone.0039800

**Published:** 2012-06-29

**Authors:** Makoto Wada, Kouji Takano, Shiro Ikegami, Hiroki Ora, Charles Spence, Kenji Kansaku

**Affiliations:** 1 Systems Neuroscience Section, Department of Rehabilitation for Brain Functions, Research Institute of National Rehabilitation Center for Persons with Disabilities, Tokorozawa, Japan; 2 Crossmodal Research Laboratory, Department of Experimental Psychology, Oxford University, Oxford, United Kingdom; French National Centre for Scientific Research, France

## Abstract

Adopting an unusual posture can sometimes give rise to paradoxical experiences. For example, the subjective ordering of successive unseen tactile stimuli delivered to the two arms can be affected when people cross them. A growing body of evidence now highlights the role played by the parietal cortex in spatio-temporal information processing when sensory stimuli are delivered to the body or when actions are executed; however, little is known about the neural basis of such paradoxical feelings resulting from such unusual limb positions. Here, we demonstrate increased fMRI activation in the left posterior parietal cortex when human participants adopted a crossed hands posture with their eyes closed. Furthermore, by assessing tactile temporal order judgments (TOJs) in the same individuals, we observed a positive association between activity in this area and the degree of reversal in TOJs resulting from crossing arms. The strongest positive association was observed in the left intraparietal sulcus. This result implies that the left posterior parietal cortex may be critically involved in monitoring limb position and in spatio-temporal binding when serial events are delivered to the limbs.

## Introduction

Adopting an unusual posture can sometimes give rise to paradoxical experiences. For example, when people cross their arms over the body midline, the subjective rank ordering of successive unseen tactile stimuli delivered to both arms can be affected (often being reversed) [1], [2]. The neural processing of bodily information has been investigated previously; for instance, vision has been shown to play a significant role in modulating perceived limb position, and the superior parietal lobule appears to play an important role in this process [3]. Meanwhile, tactile stimulation of the right hand when placed across the body midline has been shown to give rise to increased activity in the right ventral intraparietal sulcus (VIP) in participants whose eyes are closed. However, the fact that activation shifts to a left parietofrontal network when the eyes are opened, suggests that visuo-tactile multisensory limb position is likely represented in these areas [4]. The upper part of the left posterior parietal cortex is activated during the updating of limb position when people reach with their arm while their eyes are closed [5]. Recent neuroimaging studies have highlighted the involvement of the posterior parietal cortex or the temporoparietal junction when participants perform unimodal visual or tactile temporal order judgments (TOJs) [6], [7], [8]. The growing body of published evidence therefore suggests the intimate involvement of the parietal cortex in spatio-temporal information processing in humans when sensory stimuli are delivered to the body surface or when actions are executed. However, that said, little is known about the neural basis of paradoxical feelings that may result when unusual static limb positions are adopted.

In the present study, we specifically focused on the neuronal basis of the subjective reversal of tactile TOJs when the hands are crossed [1], [2] using combined fMRI and psychophysics. In doing so, activity was observed in the left posterior parietal cortex when participants adopted a crossed hands posture with their eyes closed. Furthermore, by assessing tactile temporal order judgements (TOJs) in the same individuals, we observed a positive association between activity in this area and the degree of reversal in TOJs resulting from crossing one's arms (i.e., individuals with the highest activity showed the greatest degree of reversal). These results therefore suggest that the left posterior parietal cortex is critically involved in monitoring limb position, and that the area also plays a role in predetermining subjective spatio-temporal experience when serial events are delivered to the limbs.

## Materials and Methods

### Participants

Twenty participants (all male, 19–44 years old) took part in this study. Male participants were used because sex-differences in the magnitude of the paradoxical experiences elicited when performing tactile temporal order judgments with crossed hands has been reported previously [9]. All were neurologically normal and strongly right-handed (+60≤L.Q.≤+100) according to the Edinburgh Inventory [10]. The study received ethical approval from the institutional review board of the National Rehabilitation Center for Persons with Disabilities, and all participants provided written informed consent in line with institutional guidelines.

### MR scanner task

Each participant was placed in a MR scanner with their arms uncrossed in one condition and crossed in the other. The participant's eyes were either closed (EC) or open (EO). Each participant experienced three arm positions: left over right arm (Crossed L), right over left arm (Crossed R) and arms uncrossed (Uncrossed). Each participant therefore experienced six conditions in total, with the order of presentation counterbalanced across participants. Each condition consisted of four 40 s epochs. Different auditory beeps were used to mark the start and end of each epoch. The participants were instructed to change their arm position from the rest position (outstretched beside the legs) to the test position (on the legs) with their arms either uncrossed or crossed (Crossed L or Crossed R) during each epoch. Before the task started, participants were given verbal instructions concerning the content of the task. During the experiments, the participants wore earplugs to reduce background noise, and auditory beeps and instructions were delivered via earphones (Avotec SilentScan SS3000; Stuart, FL, USA); participants' movements were visually monitored from an operator room through a window (foot side of the scanner).

### Scanning Parameters

Functional MRI data were acquired with a 1.5 Tesla MRI scanner (Toshiba Medical Systems, Tochigi, Japan). Functional images sensitive to blood oxygen level-dependent (BOLD) contrast were obtained from a T2* gradient-echo echo-planar imaging pulse sequence with a 220 mm field-of-view, 6 mm slice thickness, 2 mm interslice gap, and a 64×64 data matrix. For each session, 180 image volumes were acquired per session with a TR of 2000 ms, TE of 40 ms, and flip angle of 85°. The image volumes covered the entire brain with 20 slices.

### Data analysis

Functional images were analysed with statistical parametric mapping software (SPM8; Wellcome Department of Cognitive Neurology, London, UK) with Matlab 2007a (MathWorks, Natick, MA, USA). The image processing for each experiment was as follows: (1) motion correction; (2) co-registration of the anatomical T2 images with the mean functional images in a run; (3) spatial normalization of all images to the Montreal Neurological Institute (MNI) reference brain; and (4) spatial smoothing with a Gaussian kernel of 8 mm full-width at half-maximum.

**Figure 1:**
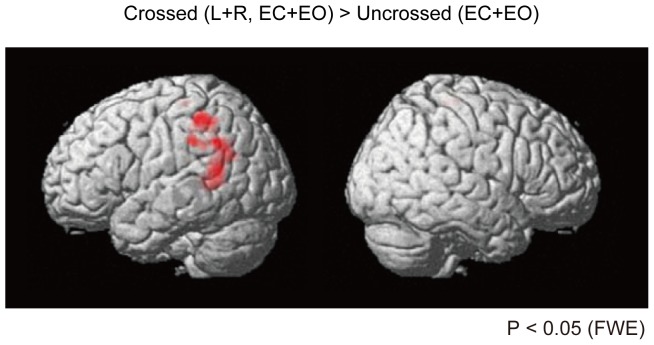
fMRI activity in the arm crossed conditions (Crossed - Uncrossed contrast). The Crossed - Uncrossed contrast was computed as [(EC Crossed L+EO Crossed L+EC Crossed R+EO Crossed R)/2−(EC Uncrossed+EO Uncrossed); *P*<0.05, FWE, extent volume >90 voxels. The contrasts were masked by the arms crossed condition at *P* = 0.05.

The statistical analysis was performed in two stages, assuming a mixed-effects design. In the first level analysis, each participant's time series was analysed separately as a fixed-effect analysis. Using the model parameters estimated by the least-mean-squares method, the resulting set of voxel values for each comparison constituted a statistical parametric map (SPM) of the *t* statistic. In the second-level analysis, all activations were isolated using a one sample *t*-test of the individual contrast as a random-effect analysis. We computed T contrasts between crossed conditions and uncrossed conditions [Crossed – Uncrossed contrast: (EC Crossed L+EO Crossed L+EC Crossed R+EO Crossed R)/2−(EC Uncrossed+EO Uncrossed); **Table 1** **and** **Fig. 1**; *P*<0.05, extent volume >90 voxels after family-wise error (FWE) corrections].

**Table 1:** Regions showing increased activity in the arms-crossed condition (Crossed L and Crossed R) compared to the uncrossed condition with the participants' eyes closed (EC) and open (EO).

Region	L/R	BA	Peak coordinates			Peak *Z*-value	*κ*_E_
			*x*	*y*	*z*		
Inferior Parietal Lobule	L	40	−42	−47	46	5.08	1430
			−46	−47	4	5.05	
Superior Temporal Gyrus		39	−44	−49	11	5.01	
Paracentral Lobule	L	6	−7	−33	57	4.83	127
	R	6	2	−33	59	4.74	
		5	9	−43	58	4.64	

*p*<0.05 (FWE), Extent volume >90 voxels, Talairach coordinates. BA, Brodmann areas; L/R, left/right hemisphere.

**Figure 2:**
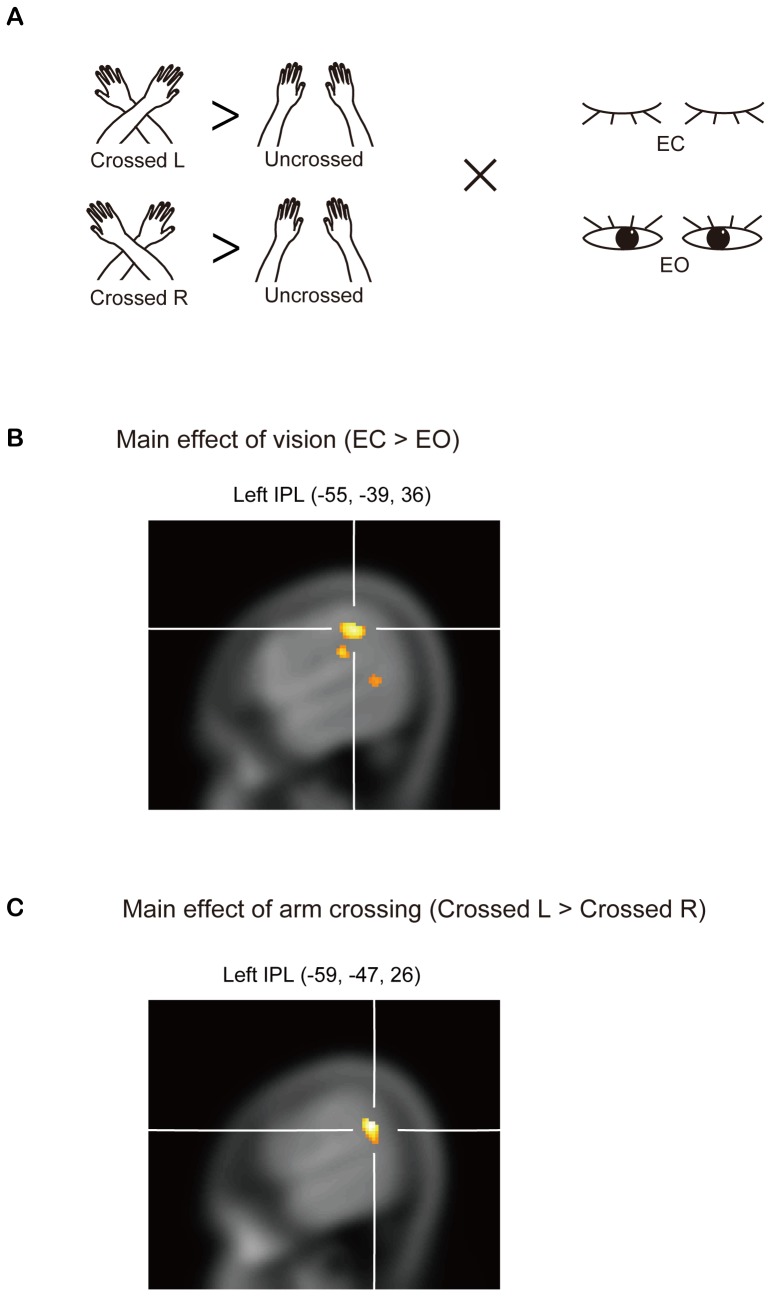
Experimental conditions and ANOVA data from the functional imaging. (**A**) Experimental conditions. Each participant experienced three arm positions: left over right arm (Crossed L), right over left arm (Crossed R) and arms uncrossed (Uncrossed). The participants' eyes were either closed (EC) or open (EO). Each participant therefore experienced a total of six conditions, with the order of presentation counterbalanced across participants. (**B**) The left inferior parietal lobule region that showed significant main effect under (EC or EO) conditions [(−55, −39, 36); *P*<0.05, uncorrected, masked by [(EC Crossed−EC Uncrossed)−(EO Crossed−EO Uncrossed) contrast at *P* = 0.05]. (**C**) The left inferior parietal lobule region that shared a significant main effect under (Crossed L or Crossed R) conditions [(−59, −47, 26); *P*<0.05, uncorrected masked by [(Crossed L−Uncrossed)−(Crossed R−Uncrossed) contrast at *P* = 0.05].

To further evaluate the effect of arm position (Crossed L, Crossed R) and eyes closed/open (EC, EO), a two-way analysis of variance (ANOVA) was performed to obtain F contrasts {[(Crossed L−Uncrossed) or (Crossed R−Uncrossed)]×[(EC Crossed−EC Uncrossed) or (EO Crossed−EO Uncrossed)]; **Fig. 2**; *P*<0.05, uncorrected}. One should be somewhat cautious given the use of the uncrorrected threshold, but we specifically focused on the left posterior parietal cortex in the ANOVA based on the results of the whole brain analyses (**Fig. 1**) and on the basis of other findings already published in the literature [3], [4], [5], [6], [7], [8].

**Figure 3:**
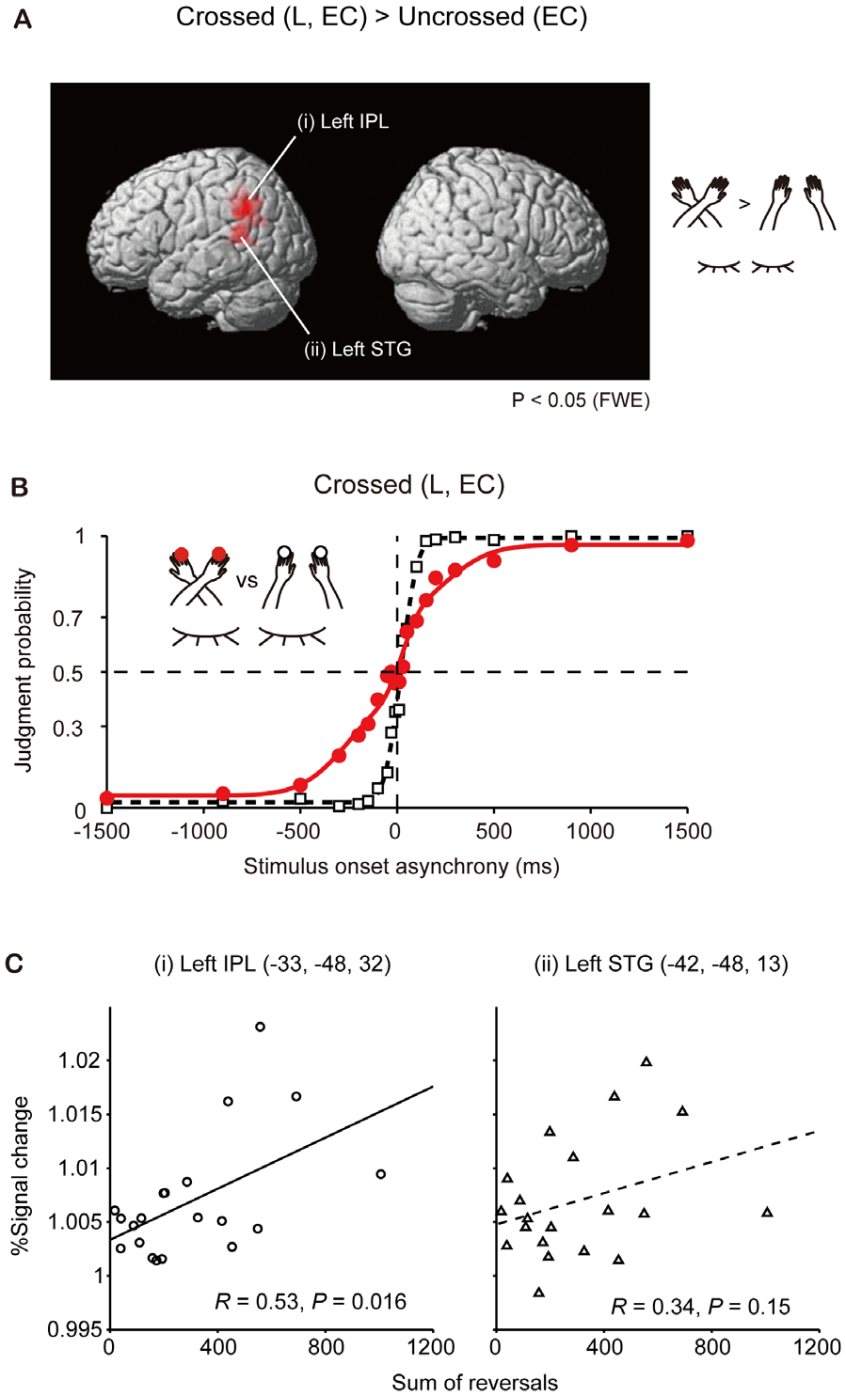
Functional imaging and behavioral data in the left-over-right-arm crossed condition with eyes closed (Crossed L, EC). (**A**) Crossed L–Uncrossed contrasts calculated using functional imaging: [(EC Crossed L)−(EC Uncrossed); *P*<0.05, FWE, extent volume >90 voxels]. The contrasts were masked by the arms-crossed condition at *P* = 0.05. (i) The left inferior parietal lobule (IPL, −33, −48, 32), and (ii) the left superior temporal gyrus (STG; −42, −48, 13). Each peak (in Talairach coordinates) was derived from statistical parametric mapping. (**B**) Tactile TOJs in the Crossed L condition (solid symbols) and Uncrossed (open symbols) conditions. The judgment probability (ordinate) that participants reported their left hand to have been stimulated second is plotted against the stimulus onset asynchrony (SOA; abscissa). The black dashed and red solid functions highlight the results of the model fitting [11] under the Uncrossed and Crossed L conditions, respectively. Each dot represents the averaged data from the 20 participants. (**C**) Panels highlighting the correlation between signal changes in each region of interest (IPL: Inferior parietal lobule, STG: superior temporal gyrus) and the sum of the reversals.

Furthermore, we computed the Crossed L – Uncrossed contrast with participants' eyes closed: [(EC Crossed L)−(EC Uncrossed); **Table 2** **and** **Fig. 3**]. The region of interest (ROI) was defined as a sphere with a diameter of 10 mm at each local peak of *P*-values in the Crossed L–Uncrossed contrast (**Table 2**; *P*<0.05, FWE) using MarsBaR software (region of interest toolbox for SPM, Brett et al., 2002). We defined ‘% signal change’ as the mean BOLD signal change in the arms-crossed conditions as compared with that in the arms-uncrossed conditions.

**Table 2:** Regions showing increased activity in the Crossed L condition, compared to the Uncrossed condition, when the participants' eyes were closed.

Region	L/R	BA	Peak coordinates			Peak *Z*-value	*κ*_E_
			*x*	*y*	*z*		
Superior Temporal Gyrus	L	39	−42	−48	13	5.10	1341
Inferior Parietal Lobule	L	40	−33	−48	32	5.04	
Superior Parietal Lobule	L	7	−26	−51	42	5.04	

*p*<0.05 (FWE), Extent volume >90 voxels, Talairach coordinates; BA, Brodmann area; L/R, left/right hemisphere.

### Psychophysics

After scanning, participants took part in the tactile TOJ task. The task consisted of two sessions: Uncrossed and Crossed L, all conducted with the participant's eyes closed. The order of the three conditions was the same as for the MR imaging. Solenoid skin contactors (Uchida Denshi, Tokyo, Japan) were used to deliver brief tactile stimulation (10 ms duration) to the dorsal surface of the ring finger of each hand. Two successive stimuli were delivered, one to each ring finger, separated by intervals randomly assigned from 20 intervals (−1500, −900, −500, −300, −200, −150, −100, −50, −30, −10, 10, …, 1500 ms). Positive intervals indicated that the participant's right hand was stimulated first and vice versa for negative intervals. The participant had to press a button with the index finger of the hand that had been stimulated second. After fMRI scanning, participants were verbally instructed about the nature of the subsequent TOJ task. In each epoch, the 20 intervals were presented in a random order. Consequently, one session consisted of 120 trials. During the experiment, the participants had to close their eyes while white noise (80 dB) was played over headphones placed over the participant's plugged ears. Data analysis was as described by Wada *et al.* [11].

The order-judgment probabilities that the right hand was stimulated earlier (or that the left hand was stimulated later) in the arms-uncrossed and arms-crossed conditions were determined using two fitting functions as described in Wada et al. [11]. First, the order-judgment probability in the arms-uncrossed condition was fitted using a cumulative density function of a Gaussian distribution [P_u_(t)]. By contrast, the order-judgment probability in the arms-crossed condition was fitted by the double flip model [P_c_(t)] [2]. Matlab (optimization toolbox) (MathWorks, Natick, MA, USA). This was used for the estimation in order to minimize the Pearson's chi-squared statistic (**Fig. 3B**).

In order to evaluate the increase in reversals caused by arm crossing, we calculated the sum of the absolute differences between the response functions of the arms-uncrossed condition and those of the arms-crossed condition for each participant (−1500 ms≤*t*≤1500 ms) and defined the sum as a ‘sum of reversals’ (*SR*) as follows.
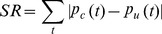
The *SR* provides a rough metric to indicate an increase in reversals resulting from arm crossing.

**Figure 4:**
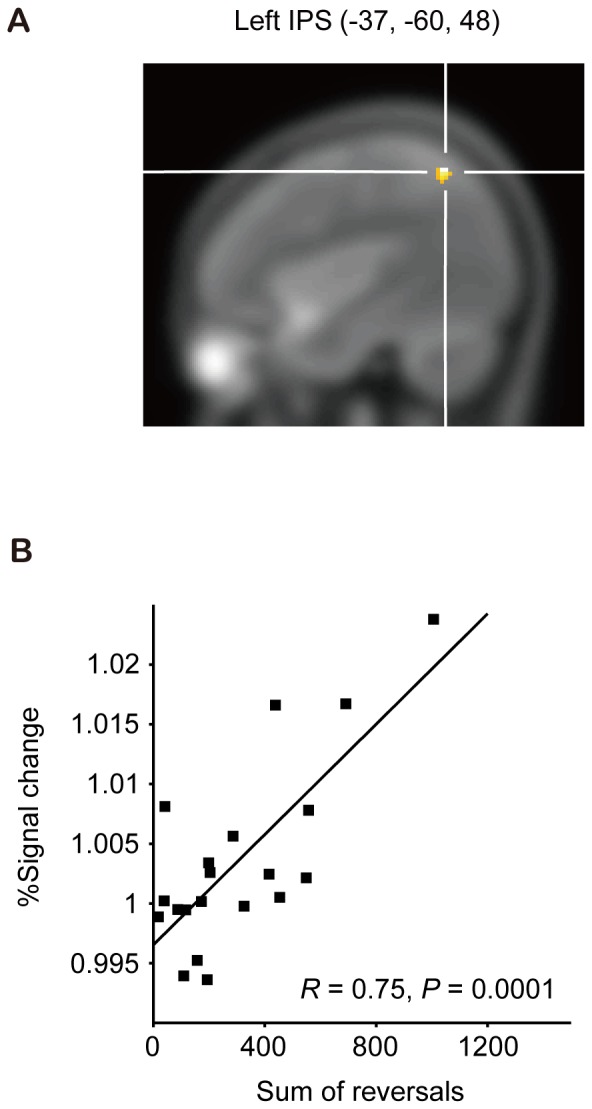
Regression analysis between the SR and the fMRI signal changes under the crossed L condition with participants' eyes closed. (**A**) The left intraparietal sulcus that showed a significant correlation between the fMRI signal changes (EC Crossed L - EC Uncrossed) and the degree of TOJ reversals due to arm crossing [(−37, −60, 48); *P*<0.01, uncorrected, masked by the Crossed L–Uncrossed contrast at *P* = 0.05]. (**B**) Panel highlighting the correlation between signal changes in the region of interest and the sum of the reversals.

We evaluated the correlation between the SR in the psychophysical experiment and fMRI results of [(EC Crossed L)−(EC Uncrossed); **Fig. 3A**] (**Fig. 3C**). We also added the regression analysis between the SR and the fMRI signal changes (**Fig. 4A** **and** **Table 3**) (P<0.01, uncorrected, extent volume >90 voxels, masked by the Crossed L–Uncrossed contrast at P = 0.05), and again evaluated the correlation between the SR in the psychophysical experiment and fMRI results of the regression analysis (**Fig. 4B**). Note that the uncrorrected threshold was used here, but remember that we specifically focused on the left posterior parietal cortex given the results of the whole brain analyses (**Fig. 3A**).

**Table 3:** Regions showing a significant correlation between the degree of TOJ reversal and fMRI signal changes in the Crossed L condition, compared to the Uncrossed condition, when the participants' eyes were closed.

Region	L/R	BA	Peak coordinates			Peak *Z*-value	*κ*_E_
			*x*	*y*	*z*		
Intraparietal Sulcus	L	40/7	−37	−60	48	3.13	93

*P*<0.01(uncorrected), Extent volume >90 voxels, Talairach coordinates; BA, Brodmann area; L/R, left/right hemisphere.

## Results

During fMRI scanning, participants were required to change their arm position from the rest position (outstretched beside their legs) to the test position (on the legs) either with their arms uncrossed (Uncrossed) or arms crossed. Given that vision of the body modulates brain activity [3], the participants in the present study took part in both an eyes closed (EC) and an eyes open condition (EO). The participants also took part in both left arm over right arm crossing (Crossed L) and right arm over left arm crossing (Crossed R) conditions, given previous findings indicating that people tend to report stronger paradoxical temporal order judgments in the Crossed L condition [6]. Note that no errors were observed when participants performed any of these tasks.

We first investigated brain activity during arm crossing. The left posterior parietal cortex and temporo-parietal junction (BA40 and 39) were activated when we compared the arm crossed conditions (Crossed L and Crossed R) to the arm-uncrossed condition (Uncrossed) under the EC and EO conditions (*P*<0.05, FWE corrected, extent volume >90 voxels, masked by the arms crossed condition at *P* = 0.05; **Fig. 1**). Activity was observed in the bilateral paracentral lobules (BA 6 and 5) (**Table 1**).

These results suggest the involvement of the left posterior parietal cortex in arm crossing (**Fig. 1**). This result is consistent with published studies highlighting the intimate involvement of the parietal cortex in spatio-temporal information processing [3], [4], [5], [6], [7], [8]. Thus, we specifically focused on the left posterior parietal cortex when examining the main effects of vision and arm crossing. The two-way ANOVA revealed a main effect of vision {[(EC Crossed−EC Uncrossed) or (EO Crossed−EO Uncrossed)]; *P*<0.05, uncorrected, masked by [(EC Crossed−EC Uncrossed)−(EO Crossed−EO Uncrossed)] contrast at *P* = 0.05; **Fig. 2A, B**}. The figure revealed stronger activation in the left posterior parietal cortex [*Z* = 2.52, −55, −39, 36 (*x*, *y*, *z*) Talairach coordinates] in the EC Crossed condition than in the EO Crossed condition (**Fig. 2B**). In addition, a main effect of arm crossing was observed {[(Crossed L−Uncrossed) or (Crossed R−Uncrossed)]; *P*<0.05, uncorrected, masked by [(Crossed L−Uncrossed)−(Crossed R−Uncrossed)] contrast at *P* = 0.05; **Fig. 2C**}. The figure revealed stronger activation in the left posterior parietal cortex (*Z* = 2.69, −59, −47, 26) in the Crossed L-Crossed condition than in the Crossed R-Crossed condition (**Fig. 2C**).

When comparing the two test conditions (arms uncrossed vs. crossed) under the Crossed L condition with participants' eyes closed (Crossed L–Uncrossed contrast), the left posterior parietal cortex (BA40) showed greater activation in the arms crossed condition than in the Uncrossed condition (*P*<0.05, FWE corrected, extent volume >90 voxels, masked by the arms crossed condition at *P* = 0.05; **Fig. 3A** and **Table 2**). Strong asymmetrical brain activity centred on the left posterior parietal cortex was observed in the Crossed L condition. Activity was also observed in the left superior temporal gyrus (**Table 2**).

After scanning, the participants took part in a tactile TOJ experiment [2]. Two brief tactile stimuli were delivered successively, one to either ring finger, separated by intervals in the range ±1500 ms. Participants responded in a forced choice manner, pressing a button with the index finger of the hand that had been stimulated second. TOJ reversals were observed when the arms were crossed (**Fig. 3B**), as has been reported previously [1], [2], [11]. In individual analyses, reversed judgments (i.e., judgment probability >0.5 and SOA <0, judgment probability <0.5 and SOA >0) were observed for 7 out of the 20 participants in fitting the data. The degree of judgment reversal was evaluated by calculating the sum of the reversals (SR), which provides a rough metric to indicate an increase in reversals resulting from arm crossing. The SR in the psychophysical experiment was found to correlate with fMRI signal changes in the left inferior parietal lobule (IPL, BA40; *Z* = 5.04, −33, −48, 32, *R* = 0.53, *P* = 0.016; **Fig. 3C**) after Pearson's product-moment correlation coefficients between the SR and fMRI signal changes had been calculated. By contrast, no significant correlations were observed in the left superior temporal gyrus (Z = 5.10, −42, −48, 13; **Fig. 3C**).

Additionally, the regression analysis between the SR and the fMRI signal changes revealed that the correlation was highest in the left intraparietal sulcus (IPS; Z = 3.13, −37, −60, 48; **Fig. 4A** **and** **Table 3**), which was more posterior and higher than the peak in the Crossed L–Uncrossed contrast (*P*<0.01, uncorrected, extent volume >90 voxels, masked by the Crossed L–Uncrossed contrast at *P* = 0.05; **Fig. 4A**). The ROI analysis of the region revealed a high correlation between the SR and fMRI signal changes (*R* = 0.75, *P* = 0.0001; **Fig. 4B**).

## Discussion

Increased fMRI activation was observed in the left posterior parietal cortex when participants adopted a crossed-hands posture with their eyes closed; subjective reversals in a crossed hands tactile TOJ task were correlated with activation in this area.

It has been reported that the posterior parietal cortex, especially the right side, is involved in the processing of spatial information [12], [13], [14]. Recent advances in neuroimaging techniques have now started to allow researchers to investigate the underlying neuronal basis of spatial information processing in humans. Pellijeff et al. [5] used an eye closed reaching task in order to highlight fMRI activations in the upper part of the left posterior parietal cortex during the updating of limb position. Meanwhile, Azanon et al. [15] applied transcranial magnetic stimulation to the right posterior parietal cortex and reported that this area played a critical role in remapping tactile stimulation onto external space. Regarding the representation of the body in space, Lloyd et al. [4] reported that tactile stimulation of the right hand, across the body midline, activated the right posterior parietal cortex including VIP, when the eyes were closed.

It has also been proposed that the posterior parietal cortex is involved in processing temporal information. The relative involvement of the right or left parietal cortices when it comes to the processing of temporal information has been much debated [8], [16], [17]. Davis et al. [7] suggested that the left temporo-parietal junction and the inferior part of the posterior parietal cortex, was involved in a “when” pathway. The participants in their fMRI study performed both a visual TOJ task as well as a shape discrimination task as a control in order to discriminate the “when” pathway from the “what” pathway [18].

Notably, the posterior parietal cortex is involved in state estimation. Lesions to the bilateral posterior parietal cortex result in postural estimation deficits [19]. Tsakiris et al. reported activation in the bilateral posterior parietal cortex during action monitoring of right finger movements [20]. A positron emission tomography study suggested involvement of the left posterior parietal cortex for dynamic estimation of hand position [21]. Furthermore, a lesion study by Wolpert et al. [22] suggested the role of this area for updating internal body representations.

The results of the present study showed increased fMRI activation mainly in the left posterior parietal cortex. Since the task used in the present study required spatio-temporal updating of the position of the arms, the lateralized activation is consistent with studies mentioned above [21], [22]. Further, observations in clinical situations have indicated that various complex or highly conceptual behaviours such as apraxic movements [23], [24] and human tool usage [25], [26] can be deleteriously affected by lesions to the left posterior parietal cortex. In terms of the representation of body parts, “Gerstmann's syndrome”, which is another condition caused by lesions to the left posterior parietal cortex [27], [28], should be highlighted here because patients with this condition suffer from left–right disorientation with regard to their body parts as well as from finger agnosis.

Lloyd et al.'s [4] study, mentioned earlier, further reported that the activation shifted to a left parieto-frontal network when their participants' eyes were open. These results therefore suggest that the multisensory representation of limb position may reside in a left parieto-frontal network. Indeed, multisensory areas, including the left posterior parietal cortex, are involved in the crossmodal binding of visual and auditory stimuli [29]. It is also known that the degree of TOJ reversal can be modified by sensory exposure such as the early development of vision [30]. Further investigations of sensory experiences may help to understand the reported individual differences in the degree of TOJ reversal due to arm crossing [1], [2], [9], [11].

One limitation of the present study is that the psychological task wasn't performed inside the fMRI scanner. This means that the activations were not induced by detecting serial stimuli delivered to the participants' limbs. Further fMRI experiments may provide useful information of a direct relationship between sensory inputs and the subjective judgment of temporal order. However, it should be remembered that the broad activation change at baseline was observed even without sensory inputs, and that the degree of activation clearly predicted the sum of reversals during the tactile TOJ. One might want to be cautious of the uncrorrected thresholds that were used in the ANOVA (**Fig. 2**) and the regression analysis (**Fig. 4**), although it should be remembered that these were analyses were based on the results of the whole brain analyses where corrected thresholds were used (**Fig. 1** **,** **3**). Applying transcranial magnetic stimulation (TMS) to check the functional relevance could help to further confirm these neuronal bases.

In summary, it is commonly believed that the superior part of the left posterior parietal cortex encodes the position of the limbs in space [5], whereas the inferior part may be more involved in the processing of temporal information [7]. The area that showed the strongest correlation between the fMRI signal change and the degree of TOJ reversal due to arm crossing in the present study was located in the middle part, i.e., especially in the intraparietal sulcus of the left posterior parietal cortex. Lesions to the left posterior parietal cortex are known to give rise to Gerstmann's syndrome [27]. The present study highlighted a broad activation change at baseline in areas in the left posterior parietal cortex resulting from the crossing of the hands, suggesting that these areas may be involved in monitoring the position of the limbs when the hands are crossed. Furthermore, the monitoring seemed to affect subjective feeling when tactile stimuli were delivered to the limbs. It has been suggested that multisensory brain areas including the left posterior parietal cortex may be involved in crossmodal binding [29]. The results reported here suggest that activity in this area may also be related to spatio-temporal binding when serial events are delivered when participants adopt an unusual posture such as crossing their arms.
